# El razonamiento clínico y la inteligencia artificial

**DOI:** 10.31053/1853.0605.v80.n4.42544

**Published:** 2023-12-26

**Authors:** Eduardo Cuestas

**Affiliations:** 1 Universidad Nacional de Córdoba. Facultad de Ciencias Médicas. Editor en Jefe de la Revista de la Facultad de Ciencias Médicas de Córdoba. Hospital Privado Universitario de Córdoba

**Keywords:** inteligencia artifical, artificial intelligence, inteligência artificial

Tanto la literatura científica como los medios de comunicación no especializados han expuesto que la inteligencia artificial (IA) podría igualar o incluso superar a

la inteligencia humana en las tareas de diagnóstico clínico.

Analizaremos aquí las diferencias en la forma en que los médicos y

la IA abordan el diagnóstico para argumentar que el razonamiento humano pervivirá y continuará siendo necesario para efectuar diagnósticos acertados, creativos y éticamente responsables dentro del contexto personal de cada paciente entendido como un ser social único e irrepetible.

Los diagnósticos de la IA se basan en algoritmos de aprendizaje automático que son teóricamente "inteligentes" como para manejar problemas difíciles y complejos. Estos algoritmos dependen a su vez de la inteligencia y de la acción humana para su creación y entrenamiento. En este campo se han logrado avances sustanciales gracias a las redes neuronales, una familia de métodos de aprendizaje automático, y particularmente de las redes neuronales profundas.

Los médicos utilizamos principalmente un enfoque hipotético-deductivo-dialéctico para realizar diagnósticos. Después de generar tempranamente hipótesis de diagnóstico, dedicamos la mayor parte del tiempo a probarlas o refutarlas recopilando más datos. Este enfoque se sustenta en procesos cognitivos que, según la teoría del proceso dual, pueden ser intuitivos o analíticos. La intuición, a veces denominada "reconocimiento de patrones", es un proceso que funciona de forma automática y subconsciente. Permite a los médicos generar hipótesis de diagnóstico tempranamente tomando algunos datos, asociándolos y comparando el resultado con patrones almacenados en la memoria a largo plazo. Estos patrones se construyen a través de experiencias de aprendizaje académico y clínico, particularmente la confrontación repetida con situaciones similares y diferentes en distintos contextos.

Hace más de 70 años que el paradigma algorítmico se pretende imponer en la práctica clínica y académica. Asumiendo que diagnosticar es sólo cuestión de "saber qué hacer en cada situación" sin tener en cuenta que los problemas que presentan los pacientes y los enfoques de diagnóstico diferencial son dependientes del contexto, de la persona y de la sociedad en que se presentan.

Esta medicina se basa en tener algoritmos de acción o normas de acción ante los problemas, por eso se ven severamente limitados desde que Kurt Gödel demostró que existen hipótesis indecidibles, es decir que ninguna teoría matemática formal es a la vez consistente (sin contradicciones) y completa (responde todas las preguntas), y que por tanto no es posible crear un algoritmo capaz de dar cuenta de todas las interrogantes en un cuerpo de conocimiento. Posteriormente, Paul Cohen, demostró que siempre que hay n axiomas es necesario uno más (n+1). Así, no todos los problemas diagnósticos de medicina clínica pueden ser resueltos por normas, manuales o computadoras y aún hay espacio para pensar, razonar y crear.

A grandes rasgos, los programas de IA toman enormes cantidades de datos, buscan patrones y se vuelven cada vez más competentes a la hora de generar resultados estadísticamente verosímiles, expresados en un lenguaje y un pensamiento en apariencia humanos. Pero sabemos, gracias a la lingüística genética y la psicología cognitiva, que estos programas difieren profundamente de la forma en que los humanos razonamos y utilizamos el lenguaje formal. Estas diferencias suponen limitaciones reveladoras en lo que pueden hacer los programas de IA, y los dotan de defectos actualmente imposibles de erradicar. De hecho, estos programas están estancados en una fase elemental de la evolución cognitiva. Su defecto más profundo es la ausencia de la mayor de las capacidades críticas de cualquier inteligencia humana: decir no solo lo que ocurre, lo que ocurrió y lo que ocurrirá —es decir, describir y predecir—, sino también lo que no ocurre y lo que podría y no podría ocurrir. Esos son los componentes de una explicación, es decir, la característica de una verdadera inteligencia.

La inteligencia humana no es, como la IA, un enorme motor estadístico de comparación de patrones que se alimenta de miríadas de datos para elaborar el diagnóstico más factible. Por el contrario, la inteligencia humana es un sistema sorprendentemente eficiente, que funciona con pequeñas cantidades de información y que no busca inferir correlaciones brutas entre puntos de datos, sino crear explicaciones. Una explicación es mucho más que descripciones y predicciones, incluye esencialmente conjeturas contrafácticas.

Como señaló Karl Popper "no buscamos teorías altamente probables, sino explicaciones; es decir, teorías poderosas y muy improbables". Las explicaciones causales son la esencia del pensamiento humano del más alto nivel. La idea de causa surge de manera intuitiva al intentar explicarnos lo que sucede a nuestro alrededor mediante un esquema lógico subyacente que nos permita relacionar unos eventos con otros mediante conexiones necesarias. El razonamiento causal es esencial en el proceso diagnóstico. La IA se basa en la descripción y la predicción, sin plantearse mecanismos causales durante el proceso diagnóstico.

Las explicaciones elaboradas por la mente humana son muy poderosas paradójicamente por su falibilidad. Para no equivocarse, debe ser posible equivocarse antes y, luego, reconocerlo. La inteligencia no solo consiste en conjeturas creativas, sino también en críticas creativas.

El pensamiento humano se basa en explicaciones posibles y en la corrección de errores, un proceso que limita gradualmente las posibilidades de errar que pueden considerarse de forma racional.

Los programas de IA tienen una capacidad ilimitada para "aprender" (léase: memorizar); sin embargo, son incapaces de distinguir lo posible de lo imposible y lo decible de lo indecible. A diferencia de los humanos que estamos dotados de una capacidad mental que limita lo que podemos aprender a aquello con un cierto tipo de complejidad genéticamente estructurada en la gramática del lenguaje que los hace inteligibles, los programas de IA "aprenden" lenguajes humanamente posibles e imposibles con la misma facilidad. Mientras que los humanos estamos limitados en el tipo de explicaciones que podemos imaginar de forma racional, los sistemas de aprendizaje automático pueden aprender simultáneamente tanto una verdad como una falsedad, pues se restringen a negociar con probabilidades que cambian simultáneamente.

Por esta razón, las predicciones de la IA son y muy probablemente serán dudosas. Como estos programas no pueden explicar las reglas del razonamiento hipotético-deductivo-dialéctico, es muy posible que predigan, erróneamente un diagnóstico por realizar una analogía desde un patrón que lo infiere erróneamente.

Las interpretaciones correctas de los síntomas y los signos expresados mediante el lenguaje humano son complejas y no se pueden obtener disfrazándolas con una enorme cantidad de datos complementarios. La tasa de errores de diagnóstico realizados por los médicos se estima entre el 5% y el 15%, según la especialidad. Se considera que los sesgos cognitivos son la causa de la mayoría de los errores de diagnóstico humano. Aunque también se han informado muchos sesgos en la literatura científica médica. El sesgo de cierre prematuro (es decir, la tendencia a dejar de considerar otras hipótesis después de llegar a un diagnóstico) se considera el más común. Otros tres sesgos comunes son el sesgo de anclaje (la tendencia a centrarse tempranamente en una o más características destacadas de la presentación inicial del problema y la imposibilidad de cambiar esta primera impresión a la luz de los datos recopilados posteriormente), el sesgo de disponibilidad (la tendencia a considerar más probables los diagnósticos que son fáciles de recordar, a menudo porque se han realizado recientemente) y el sesgo de confirmación (la tendencia considerar sólo datos confirmatorios en relación con la hipótesis generada, ignorando o subestimando los datos contradictorios).

En la mayoría de los casos, la tasa de error de la IA se puede calcular con precisión comparando los resultados proporcionados por el modelo de IA con los resultados esperados (considerados como la verdad) y su tasa es comparable a la obtenida en humanos en casos simples y estructurados. Hasta ahora ningún estudio publicado ha podido demostrar la superioridad de la IA sobre la inteligencia humana para realizar diagnósticos clínicos ni disminuir la frecuencia de los errores humanos. Los errores de la IA son altamente dependientes del proceso de "aprendizaje", generalmente una mala calidad de los datos de entrenamiento o una métrica de evaluación irrelevante.

Es esencial contar con un conjunto de datos que exprese toda la variedad de datos y las asociaciones reales entre ellos, y que no contenga ejemplos mal clasificados y no presente ningún sesgo que pueda llevar a la IA a "aprender" suposiciones falsas. Otras fuentes de errores, imprecisiones e incertidumbre podrían incluir el uso de un modelo inadecuado (por ejemplo, incapaz de representar el conocimiento a aprender) o un diseño experimental deficiente (por ejemplo, detener el aprendizaje demasiado pronto).

La verdadera inteligencia se demuestra en la capacidad de pensar y expresar cosas improbables, pero con sentido. Los buenos médicos son aquellos que saben reconocer una enfermedad por fuera del promedio frecuentista o una enfermedad frecuente que se manifiesta de forma poco habitual, mediante dos aproximaciones: una rápida, intuitiva que requiere mínimos esfuerzos cognitivos que podríamos analogar al "ojo clínico" y la otra lenta, analítica y deliberada que requiere un mayor esfuerzo consciente y que denominamos razonamiento clínico hipotético-deductivo-dialéctico.

Finalmente, la mayor y más importante diferencia es que sólo la inteligencia humana es capaz de tener un pensamiento moral. Esto significa que puede poner límites a la creatividad ilimitada de nuestras mentes con un conjunto de principios éticos que determinen lo que se debe y no se debe ser y hacer.

La inteligencia humana parece hasta ahora insustituible para el proceso diagnóstico en la clínica, pero dependerá de nosotros si lo humano permanecerá irreductible o emprenderemos un camino de poshumanización por sustitución de las máquinas con una profunda indiferencia moral, como fruto de la falta de inteligencia y sensibilidad humana. En el fondo la elección del paradigma médico está en nosotros. ¿Cuál estamos dispuestos a aceptar?

Eduardo Cuestas

Editor jefe - RFCM

